# Microstructural and Thermo-Mechanical Characterization of Cast NiTiCu20 Shape Memory Alloy

**DOI:** 10.3390/ma14143770

**Published:** 2021-07-06

**Authors:** Francesca Villa, Adelaide Nespoli, Francesca Passaretti, Elena Villa

**Affiliations:** National Research Council—Institute of Condensed Matter Chemistry and Technologies for Energy (CNR-ICMATE, Lecco Unit), Via G. Previati 1/e, 23900 Lecco, Italy; adelaide.nespoli@cnr.it (A.N.); francesca.passaretti@cnr.it (F.P.); elena.villa@cnr.it (E.V.)

**Keywords:** shape memory alloys, NiTiCu, thermoelastic martensitic transition, damping, microstructure, electron backscatter diffraction

## Abstract

Among NiTi-based alloys, one of the most promising and exploited alloys is NiTiCu, since the addition of Cu in substitution of Ni in the binary equiatomic NiTi has a significant influence on the martensitic transformation and the thermomechanical properties of the system. A high content of Cu improves the damping properties at the expense of phase homogeneity and workability. The present study focuses on an alloy with a high copper content, i.e., 20 at.%. For this specific composition, the correlation between the thermal treatments, microstructure, formation of secondary phases, and damping properties are investigated by several analyses. The microscopic observation, together with the compositional analysis, allowed the determination of four different phases in the alloy. Both the calorimetry and dynamic thermo mechanical measurements, which confirmed the high damping ability of the alloy, provided a characterization of the martensitic transition. Finally, the electron backscatter diffraction (EBSD) analysis detected the different crystallographic structures (i.e., cubic austenite, orthorhombic martensite, and cubic (face-centered) NiTi_2_) and their orientation in the different phases. Therefore, the present work aims to improve the knowledge of the role of secondary phases in the optimization of the NiTiCu20 alloy as a valuable alternative to typical alloys used for damping purposes.

## 1. Introduction

The NiTiCu ternary system derived from NiTi is among the most important and widely investigated ternary shape memory alloys (SMA). NiTiCu has several practical applications since the addition of Cu in substitution of Ni has an influence on the thermoelastic martensitic transformation (TMT) and the thermomechanical properties of the system. Indeed, the deformation of the alloy at room temperature is easiest since the TMT is shifted towards higher temperatures by the Cu addition and the material is in the martensitic state. Therefore, NiTiCu can be used in a different range of working temperatures compared to the NiTi binary alloy. In addition, the transformation temperatures are less sensitive to the composition variations and thermal cycling, hence the shape memory effect in these alloys is more stable and easier to control. Moreover, the narrower hysteresis of transformation that is observed for NiTiCu creates significant interest in actuator applications. As a result, the addition of Cu in NiTi systems can enrich possible applications and working conditions [1,2,3,4]. Generally, the Cu substitutes Ni in the 3–25 at% range. Different studies about the effect of the Cu content on the martensitic transformation path, microstructure, atomic positions, functional properties, and their improvement with respect to the NiTi system were presented [5,6,7,8,9,10]. Attention has been primarily devoted to the structures involved in thermo-elastic martensitic transformation: the austenite B2 parent phase, the monoclinic B19′ martensite for Cu content below 7.5 at.%, B2 to B19′ with the intermediate orthorhombic B19 martensite for Cu content between 7.5 and 16 at%, and B2 to B19 for a higher Cu content [1,5].

The principally investigated NiTiCu alloys are the ones with 5 at.% and 10 at.% of Cu [11,12,13,14,15]. For these systems, a synthetic vision of functional properties related to thermoelastic martensitic transformation is summarized in the following aspects: thermal hysteresis is narrower than in NiTi, thermal stability is obtained in two or three cycles, and the stress developed in mechanical strain recovery, or stress-strain measures, are lower than the one achieved in the NiTi alloy [1,2,3,4,5,6,7,8,9,10,11,12,13,14,15,16].

For these reasons, the NiTiCu alloy was principally studied for shape memory recovery and applied in actuators [3,17,18]. In recent scientific literature, interest was devoted to nano-structuration by ball milling and to preparation by sintering processes from powders [19,20,21,22]. Moreover, several studies involve theoretical calculations and simulations to correlate the macroscopic and functional properties of NiTi alloys to microstructural characteristics and to explore the martensitic path and evolution [23,24,25,26]. Concerning the damping properties, the grain size, martensite interface density, and defect structure are important internal variables and, in the case of NiTiCu, the Cu addition enhances the behavior of the NiTi system [5,27]. Most articles present in the literature are focused on the damping characteristic and internal friction evolution with respect to the Cu content, and to temperature and frequency using the dynamic mechanical analyzer (DMA). In particular, extremely high damping was found for compositions with Cu content higher than 15 at.% and particularly good results are obtained for NiTiCu20% [28,29,30,31]. Unfortunately, this high content of Cu suppresses the workability of the alloy, principally by the formation of secondary phases, and segregates [32]. The as-cast material is therefore investigated in the present study and different types of microstructures obtained after several thermal treatments are highlighted for this particular composition. Thermal analysis is the principal reference technique for the investigation of several segregation effects (not previously presented in past literature) observed after a particular high temperature thermal treatment. For damping purposes, the material in cast condition or the use of samples obtained by electro-discharge cutting are of interest, because, in this condition, the geometry of the design of damping devices can be adaptable to various kind of applications. In our work, we consider the NiTiCu20 (at.%) alloy obtained by induction melting followed by thermal treatment, as which we observe an interesting variety of distribution of secondary phases. The calorimetric characterization and microscopic observation are useful to better understand the microstructural and functional characteristics of these phases, and the possible correlation with damping performance is explored. Since, in the literature, several studies focused mainly on the evaluation and the optimization of the NiTiCu functional properties, only few works focused on the microstructural observation and secondary phases characterization and evolution regarding NiTiCu20. This work aims at filling this gap in the study of NiTi alloys with a high Cu content.

## 2. Materials and Methods

Different samples were cut from the central section of a commercial Ni_30_Ti_50_Cu_20_ (at.%) ingot obtained by induction melting (Fison Electronic Co, LTD, China). The samples were thermally treated in an argon atmosphere at different temperatures for different times and subsequently water quenched. Table 1 lists the samples and the applied thermal treatments (TQ sample corresponds to the as-cast material).

The thermal characterization was conducted through differential scanning calorimetry (DSC) and differential thermal analysis (DTA) by the differential scanning calorimeter Q200 (TA Instruments, New Castle, DE, USA) between −75 and 150 °C with a rate of 10 °C/min and the simultaneous SDT Q600 (TA Instruments, New Castle, DE, USA) between 25 and 1400 °C with a rate of 10 °C/min. The microstructure of polished samples was observed by means of the Leitz-ARISTOMET optical microscope (Leica Microsystems, Wetzlar, Germany) and SEM LEO 1430 scanning electron microscope (Zeiss, Oberkochen, Germany), and equipped with INCA Energy 200 dispersive X-Ray spectroscopy (EDS) (Oxford Instruments, Abingdon-on-Thames, UK) for the investigation of the chemical composition. The crystallographic characterization was carried out on polished samples through the electron backscattered diffraction (EBSD) analysis with a probe (Oxford Instruments, Abingdon-on-Thames, UK) mounted on the SEM microscope. The dynamic mechanical characterization (DMTA) was performed by means of a DMA Q800 dynamic thermal mechanical analyzer (TA Instruments, New Castle, DE, USA) equipped with a liquid nitrogen cooling system. The samples of approximately 3 mm of thickness and 10 mm of width were subjected to flexural loading in single-cantilever configuration. The analyses were conducted under temperature continuous variation with a rate of 2 °C/min, at a fixed strain of 0.02% at 0.5, 1, 10 and 50 Hz.

## 3. Results

### 3.1. Thermal Characterization

**Figure 1:**
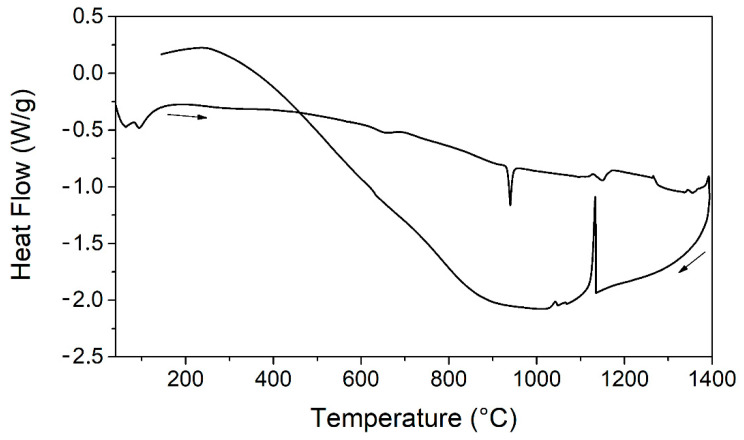
Heat flow vs. temperature up to 1400 °C for the as-cast material (sample TQ).

A preliminary characterization of the material consisted in the thermal analysis in a wide range of temperature through DTA. The evolution of the heat flow under temperature variation for the as-cast material (sample TQ) is reported in Figure 1.

**Figure 2:**
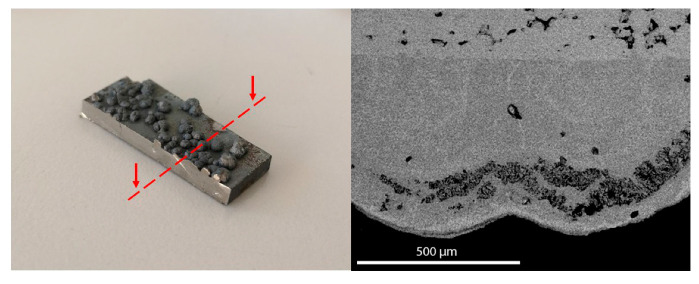
Picture of the 900C5h sample with an indication of the observed section and SEM micrograph representing the section of the segregation region.

Considering the heating route, the first endothermic peak observed between 50 and 100 °C is related to the thermoelastic martensitic transition (TMT) and is deeper investigated through differential scanning calorimetry as discussed later in this section. A second endothermic peak can be observed at approximately 930 °C and it may be related to the fusion of a eutectic compound that forms at high temperatures in the alloy with the current composition and has a lower melting point with respect to the matrix alloy. At higher temperatures, the fusion of the whole alloy occurs, and two correspondent endothermic peaks are observed in the heating route at 1130 and 1270 °C. Conversely, a single intense peak is present in the cooling route at approximately 1130 °C since the molten material solidifies in a homogeneous way. This preliminary thermal analysis enabled identification of the thermal treatment range temperatures. The chosen temperatures were 900 and 850 °C as reported in Table 1. Indeed, these temperatures right below the melting point of the eutectic compound were chosen in order to homogenize the material and maintain the original composition without element losses. It was observed that the thermal treatment at 900 °C caused the formation of rough spots on the surface, possibly caused by the rapid solidification of the molten material segregated from the matrix. The partial fusion of the eutectic phase, whose presence was revealed by the DTA measurement, may have occurred. Hence, the composition of this sample was no longer homogeneous. This was confirmed by the SEM and EDS analysis that showed the presence of voids and several regions of segregation of Cu in the bumps. Figure 2 shows an example of the segregation region and since the overall composition was not homogeneous and the structural integrity of the samples was not maintained, this sample was not further considered for mechanical measurements.

**Figure 3:**
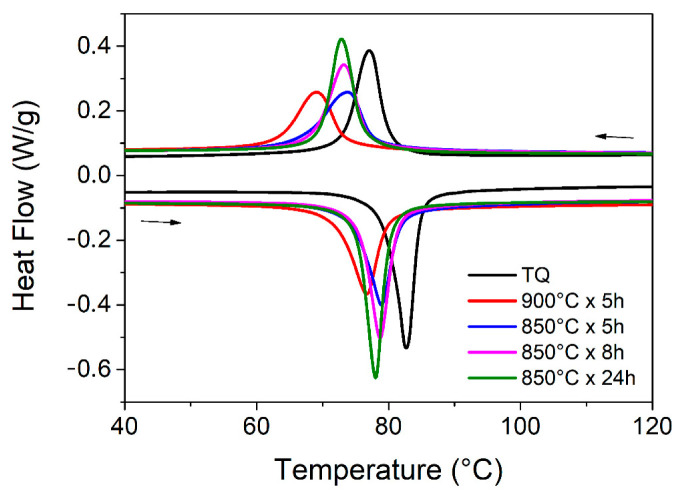
DSC analysis of NiTiCu20 samples.

Figure 3 shows the results of the DSC analysis for the as-cast and thermally treated samples, including the sample 900C5h. The peaks related to the thermoelastic martensitic transformation are visible for all the samples and the transformation temperatures and the transformation enthalpies during cooling and heating stages are displayed in Table 2.

It is possible to observe that afterward, the thermal treatments the TMT of NiTiCu20 are shifted toward lower temperatures, mostly in the case of the 900 °C treatment. Indeed, the aim of the thermal treatments at prolonged times and high temperatures is the homogenization of the alloy composition, and the shift of the transformation temperatures reveals the thermal treatment effects. Regarding treatment at a higher temperature, the DSC peaks are broader than all the others, while for the thermal treatments at 850 °C, the signals are sharper, and they increase by height with the time of the thermal treatment. As reported in Table 2, the transformation temperatures of the samples treated at 850 °C are essentially overlapping, but the transformation enthalpies increase with the thermal treatment duration.

### 3.2. Dynamic Thermal Mechanical Analysis

**Figure 4:**
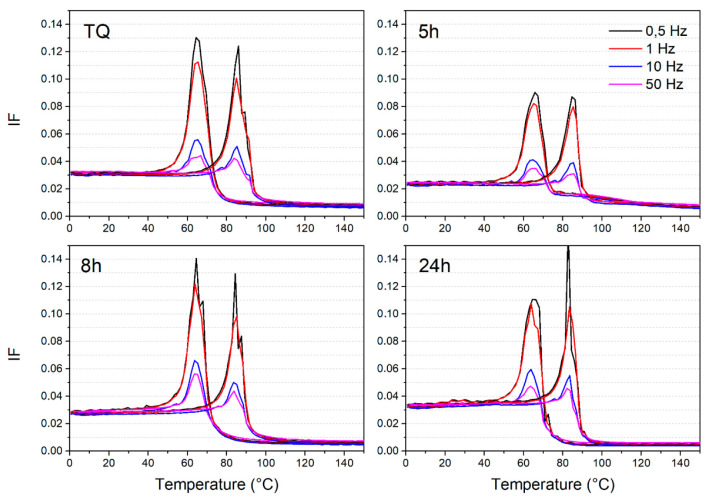
IF vs. temperature evolution for NiTiCu20 samples at different frequencies.

After the pure thermal characterization, the study focused on the investigation of the dynamic mechanical properties of this alloy that usually shows a high damping capacity, particularly ascribed to the martensitic state, which is well stable at room temperature. Figure 4 summarizes the damping behavior of the NiTiCu20 samples under temperature variation at different temperatures. As expected, high internal friction (IF) values are registered for all the samples with peaks in correspondence of the martensitic transformation up to 0.13 for lower frequencies, i.e., 0.5 and 1 Hz, and, regarding higher frequencies, the peaks are well defined and reach good values between 0.04 and 0.06. Figure 4 shows a comparison between the damping of the different samples at each frequency, and the poorer damping performance is given by the sample 850C5h, both in terms of transition peaks and the IF value in the martensitic state. Indeed, it reaches IF values of 0.09 and 0.025 at the lower frequency in correspondence of the peak and the martensitic state, respectively.

**Figure 5:**
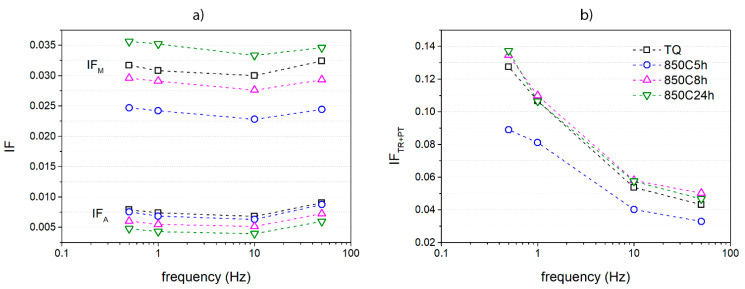
Summary of IF contributions vs. frequency for each NiTiCu20 sample in the cooling stage: (**a**) intrinsic IF values for austenite and martensite and (**b**) peak values due to the transient term and the phase transformation contribution.

The measures previously presented can be more described considering the well-known theoretical formulation of IF parameters [33]. The trends of the different contributions to IF global evolution are visible in Figure 5: both the intrinsic phase contribution of austenite and martensite respectively represented by IF_A_ and IF_M_ (Figure 5a) and the sum of transient and phase transition contributions IF_TR + PT_ (Figure 5b) are summarized. The intrinsic IF value related to austenite slightly decreases with time of the thermal treatment, while the intrinsic IF of martensite decreases with thermal treatments at lower times, i.e., 5 and 8 h, but the initial value of the TQ sample is overcome by the sample treated for 24 h. Both martensitic and austenitic intrinsic contributions slightly decrease with the frequency, but this trend presents a mild rise in correspondence to the 50 Hz frequency. In the case of IF_TR + PT_, the trend decreases with the frequency and the lowest values are given again by the sample 850C5h.

### 3.3. Microstructural Characterization

**Figure 6:**
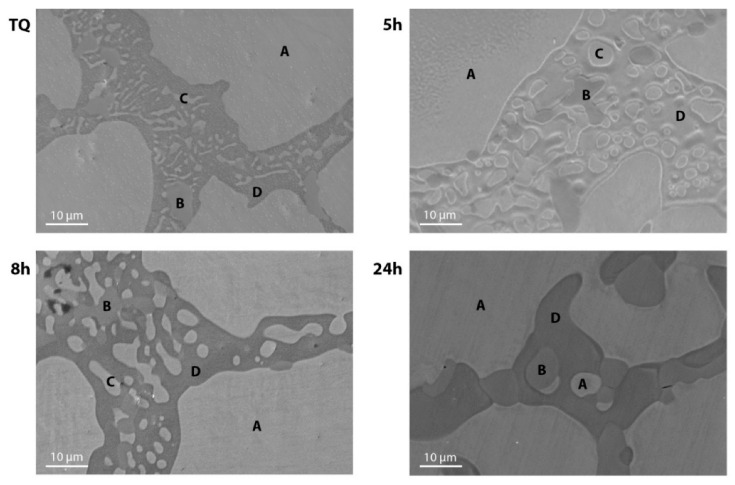
SEM micrographs of TQ and thermally treated samples at high magnification (5000×). The labels A, B, C, and D indicate the different phases identified by the EDS analysis.

In order to investigate the homogenization behavior and the microstructure of the samples, an EDS analysis was performed following the observation by means of an optical and SEM microscope. It was found that there are four phases that are present and distributed in different ways in the thermally treated samples. Figure 6 shows the high magnification SEM micrographs for the as-cast sample and samples treated at 850 °C, and the mean compositions of the phases over several measurements for each sample, by means of EDS, are reported in Table 3.

All the samples presented a matrix phase (A) which is widely spread and is mostly similar to the nominal alloy with composition Ni_30_Ti_50_Cu_20_. It was possible to observe some secondary regions spread in the samples characterized with the presence of secondary phases (labeled B, C, and D). In all the samples there is a phase, labeled as D, richer in Ti and poorer in Ni with approximately the same Cu content of 20%; this phase is spread approximately similarly in all the samples. The B phase, richer in Ti and poorer in both Ni and Cu, was identified in all the samples and it was, in some cases, distributed along almost linear paths, which may correspond to grain boundaries, as can be observed for the dark regions in Figure 4 for the sample thermally treated for 5 h. Finally, the C phase, with a slight increase of Ti and approximately the same content of Ni and Cu, changes the most in the different samples since, regarding the TQ sample, it is finely dispersed in the D phase region, while, when increasing the time of the thermal treatment up to 8 h, it seems to agglomerate in coarser regions. Instead, in the sample treated for 24 h, the absence of this phase was observed, and the observed small light regions dispersed in the D phase had the same composition of the matrix (A).

**Figure 7:**
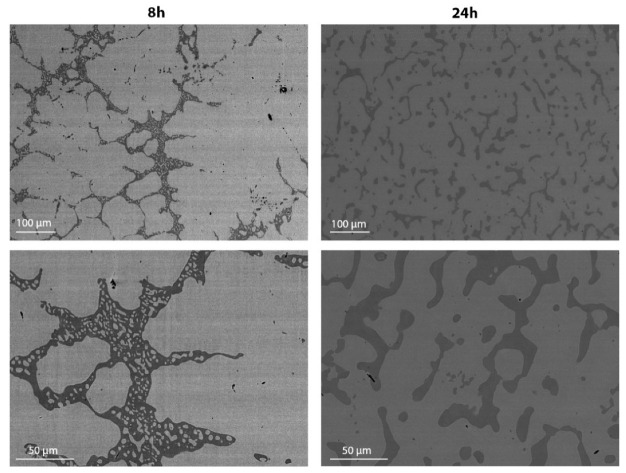
SEM micrographs at 500× (up) and 1500× (down) magnification.

It is important to notice that generally the conformation and the dimensions of the different phases seem to be constant along the same sample, but simultaneously, their distribution is not completely homogeneous. Figure 7 shows two examples of different secondary phases’ dispersion in two samples trough SEM micrographs at low magnification (500×). In the case of the region reported for sample 850C8h, the secondary phases are spread in an inhomogeneous way, while in the 850C24h sample, the dispersion of the phases is more regular.

After the identification of the different phases spread in the NiTiCu20 samples, a crystallographic characterization was performed to identify the nature of the phases. The EBSD analysis was carried out on samples 850C5h and 850C8h for their good evidence and separation of the different phases; therefore, these samples gave the suitable conditions for the EBSD analysis to identify the crystallographic structures. The observation of the samples allowed the identification of the prevalent crystallographic structures in NiTiCu20 at room temperature and a comparison between the two samples was possible. The expected predominant structure is martensite B19 since, at room temperature, all the samples are well below the thermoelastic martensitic transition. Moreover, in order to identify possible residual zones of austenite, the presence of a B2 crystal structure was investigated. The identified crystal structures and the related lattice parameters are displayed in Table 4.

**Figure 8:**
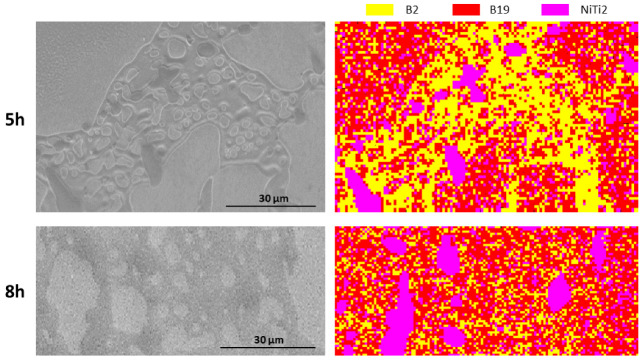
SEM micrographs of 850C5h and 850C8h samples and the related B2, B19, and NiTi_2_ crystal structures distribution identified by EBSD.

The analysis showed, for both samples, a wide presence of martensitic structure and the parent B2 austenite was detected in various regions, indicating that some residual austenite can be present, in some phases, after the martensitic transition. In addition, the NiTi_2_ cubic crystal structure was identified. Figure 8 shows the distribution of the three structures in the two samples in correspondence of the analyzed regions.

It is possible to notice that in both cases the martensitic B19 is the prevalent structure while, for sample 850C5h, it is spread preferentially in the matrix (A) and C phases, characterized by similar compositions (see Table 3). Regarding sample 850C8h, it is diffused more homogeneously within the A, C, and D phases. The presence of residual B2 austenitic phase is observed on both samples, with a higher concentration in D phase especially for the sample 850C5h. The Ti-rich B phase is distinctly characterized by the cubic crystal structure typical of NiTi_2_; considering the composition, the B phase can be considered a (Ni,Cu)Ti_2_ phase and the correspondence to the crystal structure of NiTi_2_ phase may be explained by the similarity between the atomic radii of Ni and Cu atoms. Indeed, the substitution of some Ni atoms by means of Cu ones will not significantly affect the lattice parameters [35,36].

**Figure 9:**
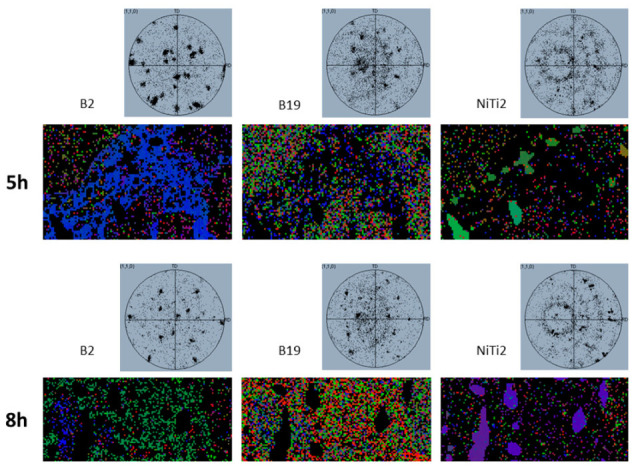
Results of the EBSD analysis performed on samples 850C5h and 850C8h. For each identified structure (B2, B19, and NiTi_2_) the pole figures (up) and the orientational maps of the normal direction with respect to the sample (down) are shown.

Figure 9 shows both the orientational maps and the pole figures of the three different crystal structures within the two samples. The pole figures revealed the presence of preferred textures for all the structures, especially for the B2 and NiTi_2_ structures. From the orientational maps in both samples, the martensitic B19 structure was spread with several orientations within phases A, C, and D. Conversely, a preferential orientation is observed in the case of the B2 structure of D phase in both samples, as revealed by the related orientational maps and pole figures, which show the evidence of textures, hence, preferred orientations. The little amount of B2 structure spread in the other phases of sample 850C5h did not show a significant preference of orientation, while in the case of sample 850C8h it seemed slightly oriented. Finally, the NiTi_2_ structure detected in correspondence of the B phase clearly exhibited preferential orientation for both samples, as evidenced by the maps and pole figures.

## 4. Discussion

The first indication about the effects of the different thermal treatments was provided by the thermal analysis. First, it was observed that the microstructural condition provided by the induction melting and its solidification structure led to the segregation of the secondary phases when the material was subjected to thermal treatment at 900 °C. Hence, the thermal treatments for NiTiCu20 have to be optimized and controlled carefully to avoid the segregation of the alloy and the subsequent loss of elements. Considering the thermal treatments at lower temperature, i.e., 850 °C, the DSC analysis showed that the main difference concerned the enthalpies related to the martensitic transformations as reported in Table 2. With an increase of the thermal treatment duration there is a slight increase of enthalpy, both in the cooling and heating stage. The as-cast sample presents enthalpies that are comparable to the samples thermally treated for longer times. Therefore, it seems that the thermal treatments cannot increase homogenization and lower the amount of defects but can only redistribute the phases with the consequence that, for some part of material, TMT is hindered. For example, for the sample 850C5h, the lower amount of martensitic structure may explain the lowering of the intrinsic value of IF at room temperature. A lower quantity of martensite may be related to a lower amount of dissipative phenomena, leading to a lower internal friction parameter. Even if, thanks to the presence of various secondary phases and corresponding defects and interfaces, the dynamic mechanical measurements shown in Figure 4 generally confirm good damping ability with high IF values typical of this alloy, the slight differences between the IF levels should be investigated. Indeed, it is not wrong to consider a more precise microstructural investigation to understand the obtained damping properties. Further investigation by the EBSD analysis gave interesting information though they did not completely align with the mechanical results. We were able to indicate the preferential structures, i.e., B2, B19, and NiTi_2_, correspondent to the different phases with their orientations, but it is evident that the EBSD is a punctual and qualitative analysis which portraits a single portion of material. Therefore, the possibility to completely correlate the crystallographic punctual information and the damping behavior is not completely feasible since the latter is provided by the combination of various defects and interfaces between the phases and inhomogeneities, which contribute to the dissipation of the vibrations. Hence, the damping can be considered a volumetric effect which combines the macroscopic contribution related to the presence of defects and interfaces and the intrinsic contribution of the crystallographic structures and cannot be easily correlated with the punctual observation of an inhomogeneous microstructure. The distribution of the different crystallographic structures in the various NiTiCu20 phases were similar for the two samples 850C5h and 850C8h in the analyzed sites. In both instances, the B19 martensite was spread within the A, C, and D phase without preferential orientations, while the residual B2 austenite detected within the D phase was strongly oriented. Regarding the NiTi_2_ structure found in correspondence of the Ti-rich B phase, there is a significant preferential orientation. The different amounts of distribution and orientation of the various crystal structures in each sample can provide different contributions to the entire damping performance that cannot be evaluated globally by the EBSD punctual analysis. However, the results of this preliminary crystallographic study are presented because there is only a small amount of similar studies in the literature [37], which are not directly related to the conditions of the current work since they regard slightly different alloy compositions and do not identify secondary phases. Despite this, the information about the different nature and distribution of the phases, their possible modulation by means of thermal treatments, and the preliminary detection of different crystal structures coexisting at room temperature can be valuable and further explored.

## 5. Conclusions

A NiTiCu20 cast alloy was investigated by means of calorimetric, mechanical, and microstructural analysis in order to explore the effects of different thermal treatments. In particular, the main outcomes are:

1. The thermo-mechanical condition of the NiTiCu20 provided by the induction melting process leads to the formation of several secondary phases which partially melt at lower temperatures with respect to the nominal alloy. Hence, the homogenization thermal treatments should be carefully optimized to avoid phases’ segregation and maintain the overall alloy composition. In the present study, 850 °C was chosen as thermal treatment temperature. The increase of the treatment time led to the increase of the enthalpy related to the TMT up to the value of the as-cast material.
2. The excellent damping ability typical of this alloy was confirmed thanks to the presence of the various secondary phases and IF reaches values up to 0.13 in correspondence of TMT. The lowest damping performance was given by the sample thermally treated for the lowest time, i.e., the sample with the lowest transformation enthalpy.
3. In order to identify and characterize the secondary phases, SEM and EDX compositional analyses were performed and four different phases were detected: a matrix phase with a nominal composition of the alloy, two Ti-rich phases with lower content of Ni and Cu, and a phase with the same content of Ti with respect to the matrix and an equal content of Ni and Cu.
4. The investigation of the crystallographic structure of the different phases allowed the detection of three structures at room temperature: the expected martensite B19, the cubic NiTi_2_ phase, and the residual austenite B2. The distribution of the structures and their preferential orientation in correspondence to the different secondary phases is identified for the samples treated at 5 and 8 h but it is difficult to correlate the punctual analysis of the crystallographic structure to the damping ability of the material, which is a volumetric phenomenon that combines defects and interfaces between the phases and intrinsic phase contributions.

## Figures and Tables

**Table 1:** Summary of the tested samples.

Sample	Temperature	Time
TQ	-	-
900C5h	900 °C	5 h
850C5h	850 °C	5 h
850C8h	850 °C	8 h
850C24h	850 °C	24 h

**Table 2:** Martensite start (Ms) and finish (Mf) temperatures, austenite start (As) and finish (Af) temperatures, and transformation enthalpies during cooling and heating stages obtained from DSC analysis of NiTiCu samples.

Sample	Ms (°C)	Mf (°C)	As (°C)	Af (°C)	ΔH_COOL_ (J g^−1^)	ΔH_HEAT_ (J g^−1^)
TQ	80.1	73.2	78.8	84.8	−9.6	11.9
900C5h	73.4	63.5	71.2	79.7	−8.0	9.6
850C5h	77.7	67.0	74.2	82.1	−9.0	10.8
850C8h	76.7	68.6	75.1	81.6	−9.4	11.2
850C24h	76.2	69.6	75.2	80.5	−9.9	11.9

**Table 3:** Compositions in at.% obtained by means of EDS of the different phases observed through SEM analysis. s.d.: standard deviation.

Phase	Ti (at.%)	Ni (at.%)	Cu (at.%)
A	50.6 (s.d. 0.2)	27.8 (s.d. 0.9)	21.6 (s.d. 0.7)
B	66.8 (s.d. 0.5)	22.3 (s.d. 1.3)	10.9 (s.d. 1.4)
C	51.9 (s.d. 1.3)	23.5 (s.d. 1)	24.5 (s.d. 1.4)
D	66.7 (s.d. 0.3)	9.7 (s.d. 0.9)	23.6 (s.d. 1.2)

**Table 4:** Summary of the crystal type and the lattice parameters of the identified crystal structures.

Structure	Crystal Type	a (Å)	b (Å)	c (Å)	α (°)	β (°)	γ (°)	Ref.
B2	Cubic (primitive)	3.015	3.015	3.015	90	90	90	[34]
B19	Orthorhombic (primitive)	2.880	4.280	4.520	90	90	90	[7]
NiTi_2_	Cubic (face-centered)	11.319	11.319	11.319	90	90	90	[35]

## Data Availability

The data underlying this article will be shared on reasonable request from the corresponding author.
